# *Bacillus* spp. characterization and his intervention as a possible non-traditional etiology of chronic renal insufficiency in Tierra Blanca, Veracruz, Mexico

**DOI:** 10.1038/s41598-020-61313-7

**Published:** 2020-03-09

**Authors:** T. A. Quiñones-Muñoz, A. M. Villares-Bueno, G. Hernández-Ramírez, R. Hernández-Martínez, M. A. Lizardi-Jiménez, V. Bocanegra-García

**Affiliations:** 1 Consejo Nacional de Ciencia y Tecnología (CONACYT) – Centro de Investigación y Asistencia en Tecnología y Diseño del Estado de Jalisco, A.C. (CIATEJ) (Centro de Investigación y Desarrollo en Agrobiotecnología Alimentaria, CIDEA). Ciudad del Conocimiento y la Cultura de Hidalgo. Boulevard Santa Catarina S/N, Santiago Tlapacoya, San Agustín Tlaxiaca, Hidalgo CP. 42163 México; 2 Tecnológico Nacional de México/I.T. Superior de Tierra Blanca, Av. Veracruz. S/N, Col. PEMEX, Tierra Blanca, Veracruz C.P. 95180 México; 3 Consejo Nacional de Ciencia y Tecnología (CONACYT) – Colegio de Postgraduados. Campus Córdoba. Laboratorio de Biotecnología Microbiana. Km. 348 Carretera Federal Córdoba-Veracruz, Congregación Manuel León, Municipio de Amatlán de los Reyes, Ver. C.P. 94946 México; 4 Consejo Nacional de Ciencia y Tecnología (CONACYT) – Universidad Autónoma de San Luis Potosí, Sierra Leona 550, Lomas 2da Secc., 78210 San Luis Potosí, México; 5 Instituto Politécnico Nacional (IPN). Centro de Biotecnología Genómica. Boulevard del Maestro. S/N, esq. Elías Piña, Col. Narciso Mendoza, Reynosa, Tamaulipas C.P. 88710 México

**Keywords:** Biotechnology, Microbiology

## Abstract

Environmental, socioeconomic, educational, custom, occupation, and native pathogen microbiota factors have been identified as unique etiological factors by region for chronic renal insufficiency (CRI). In the region of Tierra Blanca, Veracruz, there is a significant incidence of CRI. The objective of this research was to identify the presence of the genus *Bacillus* spp. and its kinetic characterization for recognition as a possible non-traditional etiology of CRI in the region. The methodology included the isolation and morphological, biochemical, molecular and kinetic characterization of strains of the genus *Bacillus* spp. and an analysis of factors that indicate that their presence could affect the occupational health of the population, prompting cases of CRI. The presence of *Bacillus cereus* (pathogenic strain for humans) was established (biochemical identification, similarity 99%, by 16S rRNA gene) in sugarcane crops, mainly in the MEX-69-290 variety, with the higher growth rate and lower lag phase, compared to the other isolates. The strains are reported as a potential danger of direct infection and a risk factor for the indirect development of CRI, in the non-traditional cause modality, in the sugarcane fields. It is recommended that committed actions be undertaken to protect and promote the health of the population.

## Introduction

Chronic kidney insufficiency (CRI) is defined as kidney damage or a glomerular filtration rate lower than 60 mL/min per 1.73 m^2^ for three months, greater urinary albumin excretion, or both^1^. 8–16% of the population worldwide is affected by CRI^2^. Hypertension, diabetes mellitus, which causes 31% of cases of CRI due to diabetic nephropathy according to Rajapurkar *et al*.^3^, urinary tract infections, obesity, and smoking, have been reported as important risk factors for the presence of microalbuminuria (an indicator of kidney injury, between 30 and 300 mg in 24 h)^4^. CRI is considered as a serious threat, as it is usually detected in an advanced state, it is irreversible, and besides increasing the possibility of death, CRI is costly to treat and consumes financial resources at an alarming rate.

In addition to the underestimation of cases of CRI, it has been reported that its incidence and prevalence, along with other diverse pathologies, differ substantially across different countries and regions^2^, indicating that environmental, socio-economic factors, education, customs, and occupation can also affect the development of pathologies. This idea has been described as the presence of unique etiological factors^5^ of non-traditional origin (ERCnt)^6^, which are specific causes by region. In fact, the WHO^7^ refers to the presence of foodborne diseases as a regional issue, not being able to report worldwide estimates regarding bacteria and chemical agents as direct causes. In addition to the already identified traditional, and more common causes, of CRI, there may be non-traditional causes (of unknown etiology) in some regions, such as herbal and environmental toxins, the presence of heavy metals, and consumption of contaminated water. Besides these possible causes, an overlap of diseases has been detected, with a prevalence of infectious diseases and an increasing prevalence and severity of disorders due to unhealthy lifestyle factors, such as obesity, diabetes, and hypertension, as well as CRI. The development of diseases overlap has been attributed to accelerated urbanization and globalization processes involved in the transformation of countries, as has been reported for South Asia and Latin America^2^.

Studies have reported that cases of advanced CRI (stage V), without identified causes, appear in younger individuals, as well as economically vulnerable individuals, compared to cases with identified causes of the disease^3^. It has also been reported that the cause or causes of kidney disease in young farmers, in productive ages, (outstanding group) in Sri Lanka and India have not been identified^5^. Other infections that cause severe kidney injury are hepatitis B and C viruses. Hepatitis C is a common comorbidity in kidney transplant recipients with end-stage renal disease, as 2.8% of kidney transplant patients were diagnosed with hepatitis C before transplantation^8^. According to Asinobi *et al*.^9^ renal disease is an important extrahepatic manifestation of hepatitis B. These authors observed a pattern of kidney disease in children (3–15 years of age) with hepatitis B, with a predominance of nephrotic syndrome, followed by glomerulonephritis non-nephrotic, end-stage renal disease, and acute kidney injury in children (24 children) seen in a hospital in southwestern Nigeria.

Kidney damage is also a complication of endocarditis, usually caused by a bacterial infection, particularly *Streptococcus* and *Staphylococcus*, of the endocardial surface of the heart^10^. Bacterial groups (called vegetations) can form and break off and travel to other parts of the body, such as the lungs, brain, abdominal organs, kidneys, and limbs, causing various serious complications, including kidney damage^11^. A study in China comprising 401 patients with infective endocarditis showed that 202 patients (50.4%) had positive microbial cultures, including *Streptococcus* (49.5%), *Staphylococcus* (28.7%), *Enterococci* (5.0%), Gram-negative Bacilli (10.9%), and others (Gram-positive *Bacillus subtilis, Candida glabrata, Leuconostoc, Pseudomonas maltophilia*, and *Micrococcus kristinae*). The rate of positive microbial cultures from patients in the renal failure group was higher than in the normal renal function group (75% *versus* 46%, *p* < 0.001). The incidence of streptococcal infection in the group with renal failure was higher than in the group with normal renal function (71.4% *versus* 43.8%, *p* = 0.001), but there were no significant differences between the two groups in the infection rates with *Staphylococcus, Enterococcus* and Gram-negative Bacilli between the two groups (*p* > 0.05)^1^.

With this background, we can visualize a possible relationship between microbial infections and the risk of developing CRI. A relationship is inferred mainly due to the presence of immunological problems that cause systemic disorders that lead to a greater development of stress (with thermal or hydric changes, which directly affect kidney insufficiency), as well as the development of diseases whose complications end in kidney damage. The genus *Bacillus* spp. are of importance, as they have been reported as opportunistic pathogens causing diseases, such as endocarditis and endophthalmitis^12^, and as already reviewed, there may be a relationship between its presence and transmission to humans, regarding the immunity and renal health.

There is experimental evidence that plants are able to control the composition of their microbiota and recruit effective protective pathogenic microorganisms in their rhizosphere or endorse^13^. Of the bacteria identified in the rhizosphere of sugarcane, *Bacillus* spp. excels. *Bacillus* spp. has the capacity to degrade hydrocarbons, such as benzopyrene^14^, and act as a bioinsecticide against lepidoptera^15^. *Bacillus* spp. can also act as a biocontrol agent, growth promoter (hormones), phosphate solubilizer, and nitrogen fixative^16^, as well as a phytopathogen^17^, and antibiotics producer for *Bacillus subtilis*^18^. The nitrification mechanisms, nitrogen fixation, facultative lithotrophy, and the acidophilic, alkalophilic, thermophilic and parasitic characteristics exemplify the ability of this genus to survive in diverse environments, as well as being able to exert diverse effects based on the context in which it is presented (e.g., substrate degrader)^19^, including the ability to cause human infections with affectations to the immune system.

In the genus *Bacillus*, the pathogens of importance regarding transmission are *Bacillus cereus*^20^*, Bacillus anthracis*^21^*, Bacillus subtilis*, and *Bacillus licheniformis*, which can be found in soil^22^, dust, and the gastrointestinal tract of animals and man. Some clinical manifestations have been identified by diarrhoeal and emetic toxins of *Bacillus cereus*, such as bovine mastitis, severe and systemic pyogenic infections, gangrene, septic meningitis, cellulitis, pulmonary abscesses, infant death and endocarditis. *Bacillus subtilis* and *Bacillus licheniformis* produce highly thermostable toxins, similar to the emetic toxin of *Bacillus cereus*^23^.

Isolates related to *Bacillus mojavensis* (REN4 and CEN2), *Bacillus amyloliquefaciens* (CEN6), *Bacillus subtilis* (CEN3), and *Bacillus cereus* (REN3 and CEN5), were obtained from the rhizosphere and endorized from plants in rice rotation (REN4 and REN3), clover (CEN6 and CEN2), and rapeseed (CEN5 and CEN3), in which antifungal activity was detected for rice crops^13^. In biocontrol^16^, the mechanism of action of *Bacillus* spp. is complex, as the genetic basis of control is not yet fully known. *Bacillus* spp. B25 is an effective control agent against the phytopathogenic fungus *Fusarium verticillioides* (Fv), with protease, glucanase, chitinase, and siderophore activities. The closely related species of the genus are *Bacillus cereus, Bacillus thuringiensis* and *Bacillus anthracis*, with a genetic relationship of 45–52%^24^.

Occupational health and environmental risk factors are considered important in the prevention of ERC nt^6^; the sugarcane activity is significant in the study region, from that point of view. Sugarcane activity has several analysis fronts: contamination of water by chemical compounds that result from the cultivation and harvesting of sugarcane, burning sugarcane and the release of its emissions to the atmosphere, and the microorganisms of the rhizosphere that are potentially pathogenic to humans. So far in 2019 (July 31, 2019), 843,650 hectares of sugar cane were cultivated in Mexico, of which 57,923,011 tons were produced. Veracruz is the state with the highest production of sugarcane in the country, which produced 7,734,477 tons so far in 2019^25^ with 85,377 hectares harvested, so far in 2019 (preliminary as of July 31). The rhizosphere of cane is an environment rich in different nutrients due to radical exudates, such as organic acids, carbohydrates, and amino acids, which makes it a rich ecosystem for the growth of a diversity of microorganisms, including bacteria^26^.

The presence of the genus *Bacillus* spp. in the root of sugarcane crops could be an indirect etiological factor of CRI, which in symbiosis with other factors, may be promoting the high incidence of the disease in the area of Tierra Blanca, Veracruz. The objective of this research was to isolate, identify and characterize strains of the genus *Bacillus* native to sugarcane fields as evidence of its presence in regions with a high prevalence of CRI and as evidence that it could be acting as one of several etiologies of the disease.

## Results and discussions

### Isolation of native strains

The Ingenio La Margarita S.A. of C.V., provided three different varieties of cane plants (COLMEX, CP-72-2086, MEX-69-290), of which the geographic location was registered (COLMEX, Lote Tempranero-Guapinole, coordinates N18°30′47.3″ W096°26′08.1″; CP-72-2086, Lote Tempranero-Buena Vista, coordinates N18°30′40.2″ W096°26′58.5″; MEX-69-290, Lote Tardío-Guapinole, coordinates N18°30′50.8″ W096°26′31.9″). Two of these varieties (CP-72-2086 and MEX-69-290), according to a technical report by SAGARPA^27^, are the most used in the country, covering 65% of the national surface. Thus, these varieties are representative of the type of cane produced in the country.

After a visual inspection of the sugarcane seedlings, it was determined that they were healthy, and no pests were present. Once the health of the seedlings was verified, portions of the root were removed, while avoiding fracturing the plant and having as little direct contact with them as possible. The cultures obtained from the root of the plants presented colonies with morphological characteristics belonging to the genus *Bacillus* spp., such as a dry or creamy consistency, as wells as a concentric ring and irregular border in the center, as described by Calvo and Zúñiga^28^ and Larrea-Izurieta *et al*.^17^. We obtained four different isolates, three from the MEX-69-290 (MEX-01-A, MEX-03-C, MEX-04-C) and one from CP-72-2086 (CP-01-C). No isolates were obtained from the seedling of the COLMEX variety. García *et al*.^29^ sampled the rhizosphere of sugarcane from different areas of the state of Tamaulipas, where 121 bacterial strains were isolated, of which 19 strains presented morphological characteristics to the genus *Bacillus* spp. Additionally, the authors reported that most of the samples were obtained from fields with old agricultural management (50 years).

### Kinetic characterization: growth kinetics

The determination of radial growth, *in vitro*, for microorganisms is considered subjective and dependent on the subject who performs the experiment; thus, the calculation of kinetic parameters is recommended to obtain more reliable data. Therefore, an adjustment was made to the Gompertz model and the corresponding kinetic parameters were determined.

We sought to obtain growth description parameters of the isolated strains to support the determination of concentrations and times considered to be of danger to humans, which could result in infections that endanger health and life. In addition, the identification of growth phases, according to previous data^16^, can indicate the type of danger that can occur (for example, *Bacillus cereus* is enterotoxic in the exponential phase of growth^12^). The high μ_max_ values indicate a higher growth rate, and therefore a higher microorganism concentration in the medium, which would increase the risk of infection. Besides being able to identify risks, determining growth curves allow for the establishment of culture conditions for the use of metabolites, such as enzymes or antibiotics produced by the isolates. In the case of the genus *Bacillus* spp., which has pathogenic microorganisms, to understand its growth curves, together with thermal death curves, will support the development of pasteurization and control conditions, or technologies, to avoid economic losses due to its capacity to produce heat-resistant spores and its ability to grow in refrigerated foods^16^.

The initial cell count was performed in the Neubauer chamber, obtaining a concentration of 1 × 10^7^ cells/mL, for inoculum. The subsequent counts, which were performed every 2 h for 12 h of the growth under the established culture medium, showed results that were analyzed using the Gompertz mathematical model. The culture medium was supplemented with sugarcane bagasse, molasses, and yeast extract (as a nitrogen source) to observe how the strains behave in a medium containing material similar to that found naturally in the sugarcane fields and to consider the nutritional needs of the genus. Using the formulated medium, the basic needs of the microorganism were complete for its growth and characterization. The limitation of nutrients can cause a decoupling of catabolic and anaerobic processes that would decrease the yield of the biomass, as well as modify the cellular physiology.

**Figure 1:**
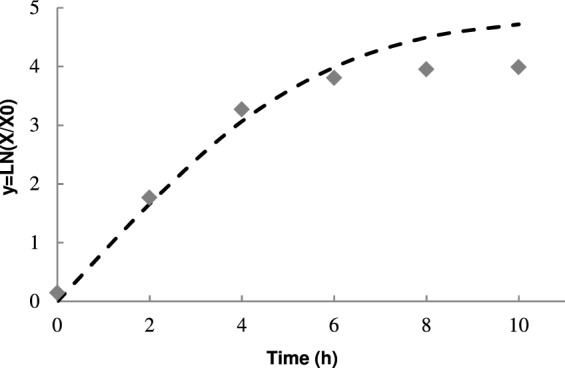
Growth of the MEX-03-C strain () in sterile water supplemented with 1% molasses, 1% yeast extract and 2% bagasse of cane. A 2-h count was performed with a Neubauer chamber (viable cells/mL). The growth data obtained were adjusted to the Gompertz model (━), to obtain the corresponding kinetic parameters (μ_max_, λ).

In the supplemented medium, growth was faster compared to growth in a standard medium (only sterile water). Isolates MEX-01-A and MEX-03-C showed the highest μ_max_ (0.99 h^−1^ and 1.03 h^−1^, respectively) and the lowest lag phases (0.37 h^−1^ and 0.27 h^−1^, respectively), which makes them potent pathogens, with a greater potential to grow in shorter times (Table 1). The inflection times determined by modeling (Ti) (Fig. 1), indicated the strains that reached the end of the logarithmic phase in less time were MEX-01-A (1.71 h) and MEX-03-C (1.70 h), which could signify the time needed to produce toxic substances, as well as the time of greatest risk of toxicity. Espinoza^30^ reported growth kinetics of *Bacillus subtilis* in Schaeffer medium supplemented with glucose, in which the microorganism presented a μ_max_ of 1.76 h^−1^ and a lag phase of 0 h (aerobic condition). The differences found between the kinetic parameters can be due to the type of medium (carbon and nitrogen sources) in which the kinetics were developed, if the carbon source is easy to assimilate or not, or the temperature, variables that directly influence the registered results. *Bacillus subtilis* has been reported as one of the species with the highest rates of growth and death^16^.

**Table 1:** Growth kinetic parameters in simple and supplemented culture medium (2% sugarcane bagasse, 1% yeast extract, and 1% molasses).

Strain	Simple growing medium			Supplemented culture medium		
	Lag (h)	Ti	µ_max_ (h^−1^)	Lag (h)	Ti	µ_max_ (h^−1^)
MEX-01-A	0.47	2.29	1.01	0.37	1.71	0.99
MEX-03-C	0.00	1.92	0.77	0.27	1.70	1.03
MEX-04-C	0.09	2.31	0.83	0.73	2.54	0.91
CP-01-B	0.75	3.25	0.88	0.50	2.29	1.03

Lag (h): Lag pase time, in hours.

Ti: Inflection time, in hours.

µ_max_ (h^−1^) = Specific maximum growth rate, in 1/hour (h^−1^).

### Biochemical identification

Biochemical tests were performed on the strains with morphological features characteristic of the genus *Bacillus* spp. and compared with the antecedents^31–33^. The results indicate a positive behavior to the gender. Table 2 shows the results for each strain as well as the base reference. All strains isolated and analyzed based on morphology tested positive for the genus *Bacillus* spp.

**Table 2:** Biochemical tests for bacterial strains.

Isolated strain	Gram staining	Catalase	Methyl red	Voges- Proskauer	Citrate reduction	Growth in NaCl 7%	Starch hydrolysis
Gender reference *Bacillus* spp.	**+**^46^	**+**^32^	**+**^47^	**+**^17^	**+**^42^	**+**^28^	**+**^43^
MEX-01-A	**+**	**+**	**+**	**+**	**−**	**+**	**+**
MEX-03-C	**+**	**+**	**+**	**+**	**−**	**+**	**+**
MEX-04-C	**+**	**+**	**+**	**+**	**−**	**+**	**+**
CP-01-B	**+**	**+**	**+**	**+**	**−**	**+**	**+**

A declaration of identification by species requires molecular analysis. *Bacillus cereus* and *Bacillus thuringiensis*, are biochemically identical, differing only in the production of the parasporal crystal (BBL CRYSTAL). Therefore, the biochemical tests were not definitive for determining the species of the strains; although, they have presented broad characteristics for the genus^34^.

### Molecular identification

DNA extraction and quantification were performed on the kinetic-identified strains as the most dangerous to the nearby population (latent pathogens). Both the sufficiency and purity of the samples were verified before continuing with molecular identification of the morphologically and biochemically identified strains of the *Bacillus* genus. Electrophoresis showed bands of DNA from each of the strains that corresponded to the V4 region of the 16S rRNA gene that we wished to amplify, with 510 bp. When the molecular weight patterns of strains show different bands, this indicates different molecular weights, a characteristic called polymorphism^34^.

The isolated bacterial strains had a high percentage of similarity in the BLAST (Basic Local Alignation Search Tool) search of the NCBI (National Center for Biotechnology Information) with the corresponding sequences available (Table 3) Program BLASTN 2.8.1^35,36^). Strain MEX-01-A presented the highest quantity of nucleic acids with an optimum purity for its replication. Based on the results, with 99% similarity it was confirmed that the isolated bacterial strains correspond to the *Bacillus* genus, with *B. anthracis* and *B. cereus* being the microorganisms identified for that strain (Table 3). The presence of *Bacillus anthracis* for the strains MEX-01-A and MEX-03-C has been found with high similarity (99% for both). More than 99% sequence similarity of the primary structure of 16S rRNA has been recorded between *Bacillus cereus, Bacillus anthracis, Bacillus mycoides*, and *Bacillus thuringiensis*. Based on the similarity between them, including phenotypic and genotypic properties, all have been considered *Bacillus cereus* varieties^12^.

**Table 3:** Molecular similarity of isolated strains and Gen Bank records (the largest Query Coverage (%) are presented).

Strain	Closest Match (Description)	Max score	Total score	Query Coverage (%)	E value	Identity	Accession No.
MEX-01-A	*Bacillus anthracis* 16S ribosomal RNA gene, partial sequence	795	795	95%	0.0	99%	KP126519.1
	*Bacillus cereus* partial 16S rRNA gene, isolate BCsn	795	795	95%	0.0	99%	HE660034.1
MEX-03-C	*Bacillus cereus* strain ST06 16S ribosomal RNA gene, partial sequence	880	880	100%	0.0	99%	MF496242.1
	*Bacillus anthracis* strain Larisa 16S ribosomal RNA gene, partial sequence	880	880	100%	0.0	99%	MH135297.1
MEX-04-C	*Bacillus cereus* strain YB37 16S ribosomal RNA gene, partial sequence	187	187	61%	3e-43	77%	KJ720024.1
	*Bacillus thuringiensis* strain 3S1^−1^ 16S ribosomal RNA gene, partial sequence	182	182	61%	1e-41	76%	MG738339.1
CP-01-B	*Bacillus* sp. EF4A-B854 16S ribosomal RNA gene, partial sequence	861	861	80%	0.0	99%	KC545277.1
	*Bacillus* sp. RKBH-B153 16S ribosomal RNA gene, partial sequence	861	861	80%	0.0	99%	JX317730.1

*Bacillus cereus* is commonly found in the environment and has the potential to contaminate food due to poor manufacturing practices (in processing or at home)^20^. *Bacillus cereus* can cause diarrhoeal and emetic syndrome with concentrations ranging from 10^3^–10^7^ cells or spores, although the exact dose response has not yet been established as described by Santa María *et al*.^37^*. Bacillus cereus* AMSB3 has also been reported as an Manganese solubilizer according to Sanket *et al*.^38^, together with *Bacillus nealsonii* AMSB4, *Enterobacter* spp. AMSB1, and *Staphylococcus hominis* AMSB5.

*Bacillus cereus* has been recognized as a bacterium of clinical importance, not only as an environmental pollutant, as it can spread to any system of food production and processing, easily infecting humans and causing affectations of health and deterioration of the immune system, along with all the consequences. Its ability to form endospores gives it the advantage of surviving in extreme conditions, such as high temperatures, even during pasteurization; it produces seven types of toxins, cereulide (emetic toxin), three enterotoxins (hemolysin BL or HBL, non-hemolytic or NHE and enterotoxin T or EntT; responsible for emetic and diarrhoeal syndrome), and three phospholipases (poisoning generators). Additionally, its affectations may not be so well identified due to the high similarities in the symptoms it causes with other strains of the genus, such as *Bacillus thuringiensis*, which is widely used as an insecticide in agriculture^12^.

From the results obtained, a pathological danger is identified in sugarcane fields that could be indirectly causing the development of diseases, such as CRI, through mechanisms of infection, immunological problems, and symbiosis with other etiologies of the disease. The presence of *Bacillus cereus* in MEX-69-290, one of the most cultivated of sugarcane varieties, indicates a strong possible effect on labor in the fields.

The presence of *Bacillus cereus*, recognized as a pathogenic strain, has been established in sugarcane crops in the region of Tierra Blanca, Veracruz, mainly in the MEX-69-290 variety, which is one of the most sown and harvested in the State of Veracruz and the country. Morphological and biochemical analyses confirmed that the isolated strains belong to the genus *Bacillus*. Strains of *Bacillus* spp. isolated from the cane root variety MEX-69-290, presented a higher growth rate with respect to the CP-72-2086 variety; so, they are reported as a potential danger of direct infection and an indirect risk factor for the development of CRI in the non-traditional cause modality. Two strains were identified molecularly as *Bacillus cereus*, with a 99% similarity rate, and four as part of the genus with similarities from 76%, between the sequences detected by the amplification of the 16S rRNA gene and those in the Gen Bank database.

## Methods

### Sampling

The plants were donated by “Ingenio La Margarita S.A. of C.V.” from the fields of the same processor. Three plants from different sugarcane varieties were randomly collected and their geospatial location (longitude and latitude) was recorded.

### Bacteria isolation

1 g of the root of the sugarcane seedlings was taken and added to tubes with 9 mL of peptonated water in a 1/10 ratio (w/v), incubated at 65 °C for 24 h, and then transferred to 4 °C for 20 min (thermal shock to control the growth of genera other than *Bacillus* spp.). Serial dilutions were made from 10^−1^ to 10^−5^, of which the 10^−1^, 10^−3^, and 10^−5^ dilutions were seeded by stria onto plates with nutritive agar. The cultures were maintained at 35 °C for 24 h. The colonies with different morphologies were isolated from the culture to obtain unique strains with homogeneous morphological characteristics based on the appearance of the shape, edge, surface, size, consistency, color, and elevation. A number was assigned to each strain isolated from the cane seedlings for their correct identification (modified from Carreras^39^).

### Morphological characterization of the isolated bacteria

The morphological characterization of strains for the genus *Bacillus* spp. was carried out based on the following: Colonies with irregular shape, serrated or finger-like borders, whitish or cream, acuminate or flat elevations^28^, and floury, waxy, dry or creamy appearance^17^.

### Bacteria biochemical characterization

Considering the strains with positive morphological tests for the genus *Bacillus* spp., the following confirmatory tests were performed for the biochemical identification of the genus: Gram-positive [Gram stain ^(31)^], catalase positive^17^, Voges Proskauer-Methyl Red (MR-VP) positive^40^, citrate positive^41^, Triple Sugar Iron Agar test, Starch hydrolysis^17,42^, survival at 7% in NaCl^43^, and Anaerobiosis (−/+) survival at 50 °C^28^.

### Kinetic characterization of strains by growth curves

A hoe was taken from each strain and inoculated into the selected culture media, to measure growth: (1) simple sterile water and (2) sterile water supplemented with 1% molasses, 1% yeast extract and 2% bagasse of cane. The samples were incubated at 35 °C for 24 h. Next, a suspension was prepared, where 250 mL Erlenmeyer flasks containing previously sterilized media (121 °C for 15 min) were inoculated with 1 × 10^7^ cells/mL, and the cells were maintained at 35 °C, with agitation/aeration. A 2-h count was performed with a Neubauer chamber (viable cells/mL). The growth data obtained were adjusted to the Gompertz model, to obtain the corresponding kinetic parameters (μ_max_, λ). This mathematical model was designed to describe growth, which helps interpret a phenomenon under controlled conditions. The model is expressed with the reparametrized Gompertz equation for sigmoidal growth by mathematical parameters, a, b, c (Eq. 1), where the specific maximum growth rate (μ_max_) is defined as the tangent at the inflection point (Eq. 2). The lag phase (λ) is defined as the intercept of the x-axis in the tangent (Eq. 3)^44^.

1 $$y=A\,exp\left\{-\exp \left[\frac{{\mu }_{max}e}{A}(\lambda -t)+1\right]\right\}$$

2 $${\mu }_{max}={(dy/dt)}_{{t}_{i}}=\frac{ac}{e}$$

3 $$\lambda =\frac{(b-1)}{c}$$

### Molecular identification of microorganisms

#### Strains propagation

The microorganisms were stored in tubes containing sterile water, under refrigeration. For activation of the strains, each bacterial strain was plated on tryptic soy agar (TSA) (35 °C for 24 h) to obtain pure colonies.

#### Obtaining DNA

The Wizard® Genomic DNA purification kit was used according to the manufacturer’s instructions. The quantification (ng/μL sample) and evaluation of nucleic acid purity was performed using a spectrophotometer (UV-vis) NanoDrop by placing 2 μL of sterile water as the target and 2 μL of the samples^45^.

#### Molecular identification

Polymerase chain reaction (PCR) was carried out in an Applied Biosystems® thermocycler. For amplification of the V4 region of the 16S rRNA gene, the mixture contained: 5.63 μL of water, 3 μL of 5X buffer, 1.1 μL of 50 mM magnesium chloride (MgCl_2_), 0.4 μL of dNTPs, 0.9 μL of PF and PR, 0.07 μL of Taq polymerase, 3 μL of the bacterial lysate (isolated DNA), in a final volume of 15 μL. The following settings were used for the amplification program: a denaturation cycle of 4 min at 95 °C, followed by 30 cycles of denaturation for 30 s at 95 °C, an alignment cycle of 30 s at 53 °C, an extension cycle for 30 s at 72 °C, and a final extension for 5 min at 72 °C^34^. To purify the amplified product, the Kit Wizard® SV Gel and PCR Clean-Up System was used according to the manufacturer’s instructions. Sequencing was performed in Eurofins Genomics (https://www.eurofinsgenomics.com/en/home/), and the sequences obtained were analyzed using the NCBI database (https://www.ncbi.nlm.nih.gov/).

## Data Availability

The datasets generated during and/or analyzed during the current study are available from the corresponding author on reasonable request.
